# A Pseudotumoral Goiter Revealing the Fibrous Variant of Hashimoto’s Thyroiditis: A Case With Multidisciplinary Diagnostic Challenges

**DOI:** 10.7759/cureus.104496

**Published:** 2026-03-01

**Authors:** Kholoud Zahouani, Mohammed El Magroud, Anass Haloui, Nassira Karich, Amal Bennani

**Affiliations:** 1 Department of Pathology, Faculty of Medicine and Pharmacy, Mohammed VI University Hospital, Mohammed First University, Oujda, MAR; 2 Department of Anatomopathology, Faculty of Medicine and Pharmacy, Mohammed VI University Hospital, Mohammed First University, Oujda, MAR

**Keywords:** anatomical pathology diagnosis, autoimmune thyroiditis, compressive goiter, diffuse thyroid fibrosis, fibrous variant, oncocytic metaplasia, squamous metaplasia, thyroid malignancy mimic, thyroid pseudotumor, hashimoto’s thyroiditis

## Abstract

The fibrosing (or fibrous) variant of Hashimoto’s thyroiditis is an uncommon entity that may closely mimic a malignant thyroid tumor due to its clinical presentation and morphological features. We report the case of a patient with progressively worsening compressive goiter in the setting of established hypothyroidism, in whom imaging findings suggested a pseudotumoral lesion. Histopathological examination of the thyroidectomy specimen demonstrated diffuse thyroid fibrosis associated with a dense lymphoplasmacytic infiltrate forming lymphoid follicles, along with oncocytic and squamous metaplasia, without evidence of malignancy. This case highlights the pivotal role of thorough histopathological assessment in establishing the correct diagnosis of this rare condition and in excluding neoplastic processes, thereby preventing unnecessary aggressive management.

## Introduction

The fibrosing (fibrous) variant of Hashimoto’s thyroiditis is an uncommon entity, accounting for less than 10% of reported cases, and it frequently remains underrecognized because of its misleading clinical and morphological features [1,2]. It is defined by extensive thyroid fibrosis that imparts a markedly firm consistency to the gland, sometimes accompanied by a rapid increase in thyroid volume and compressive manifestations. This presentation may closely mimic an aggressive neoplastic process, leading to diagnostic confusion [3].

## Case presentation

**Figure 1:**
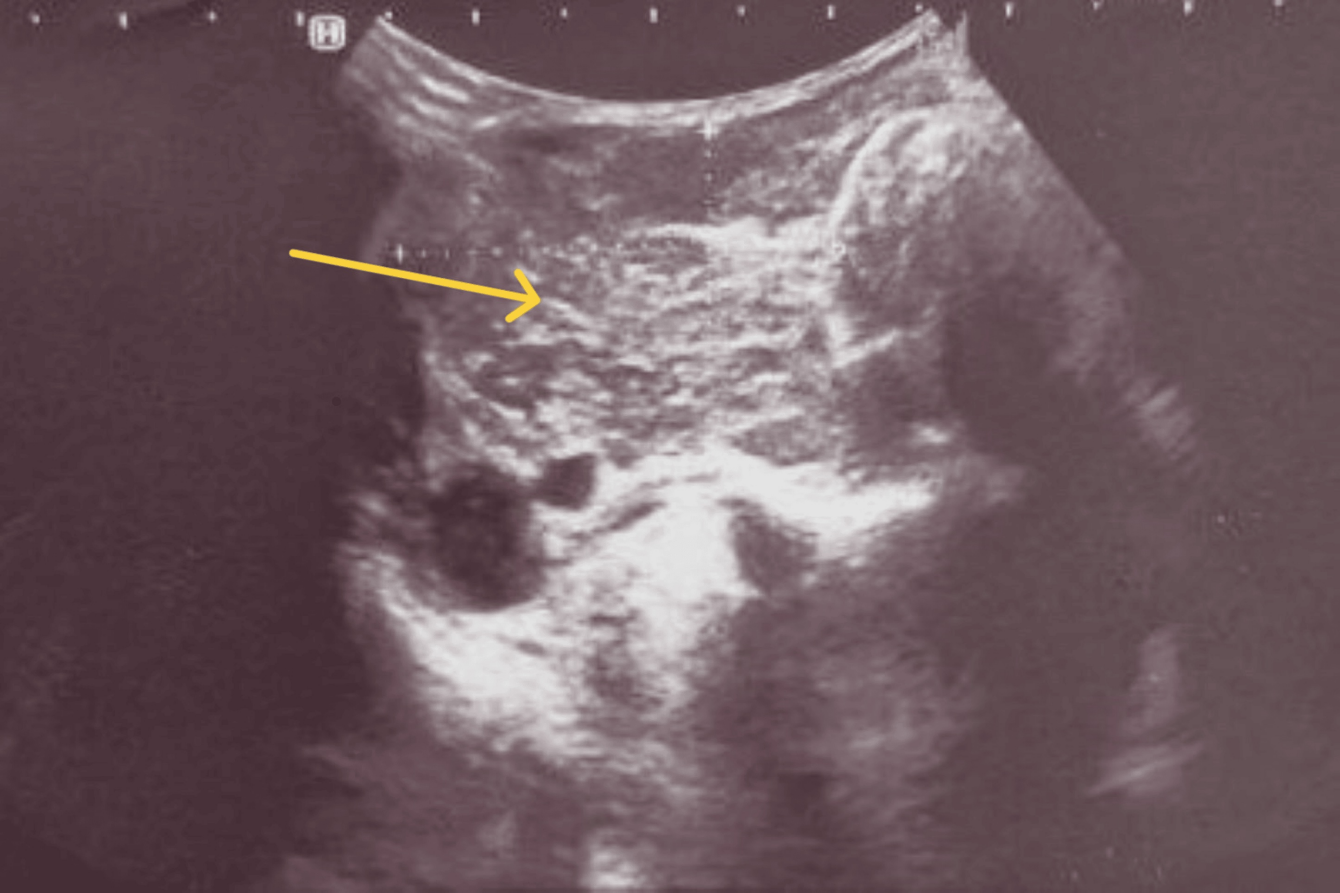
Cervical ultrasonography demonstrated a large substernal goiter with a markedly heterogeneous, multinodular architecture (yellow arrow).

A 54-year-old man, an active smoker who had been followed for hypothyroidism for two years, presented with gradually progressive dyspnea, correlating with the increase in volume of a plunging goiter. On clinical examination, the cervical mass was firm, painless, and remained mobile on swallowing. No fine-needle aspiration cytology of the thyroid was performed. Cervical ultrasonography demonstrated a large substernal multinodular goiter with marked parenchymal heterogeneity (Figure 1)

**Figure 2:**
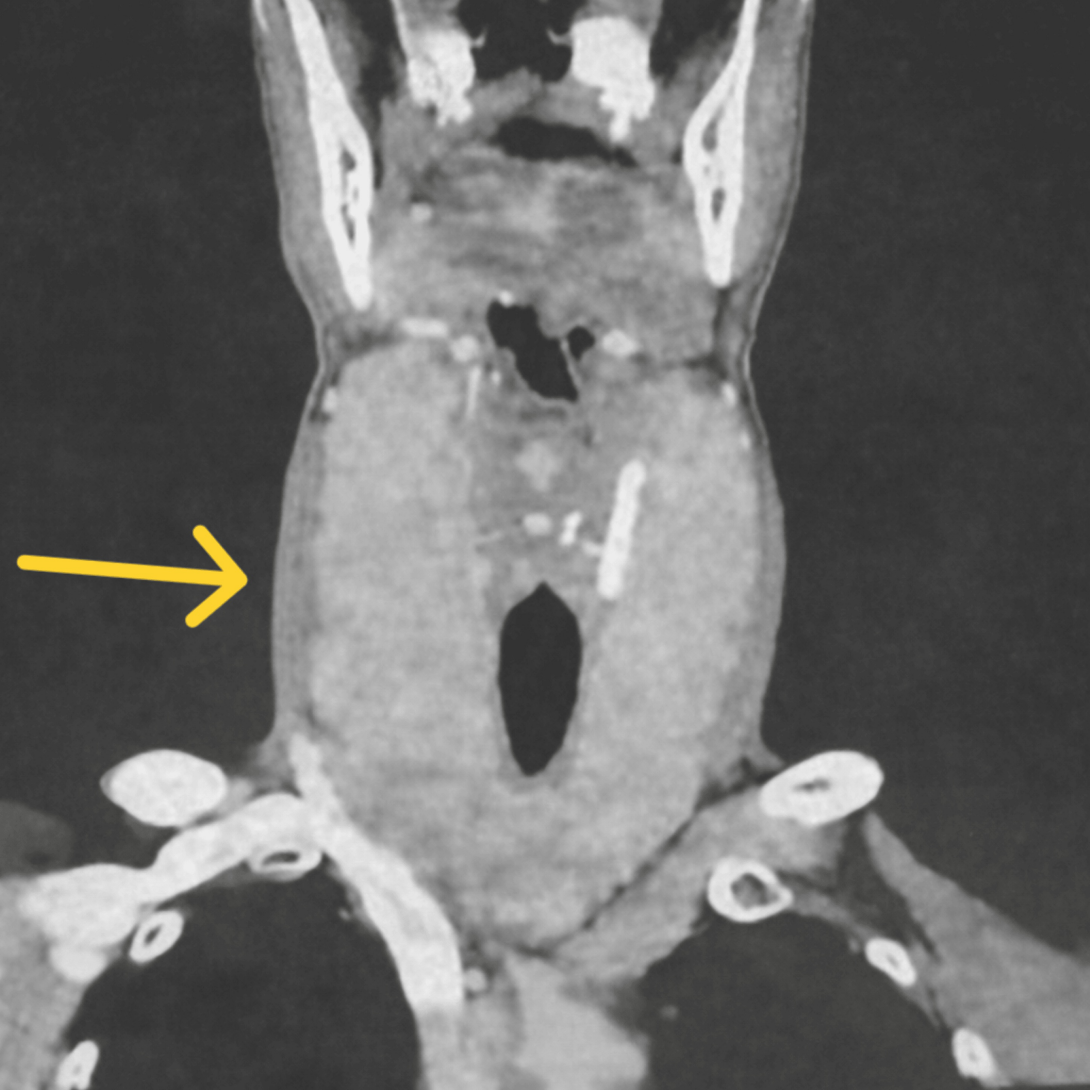
Cervicothoracic CT (frontal view) showing a large substernal goiter (yellow arrow).

**Figure 3:**
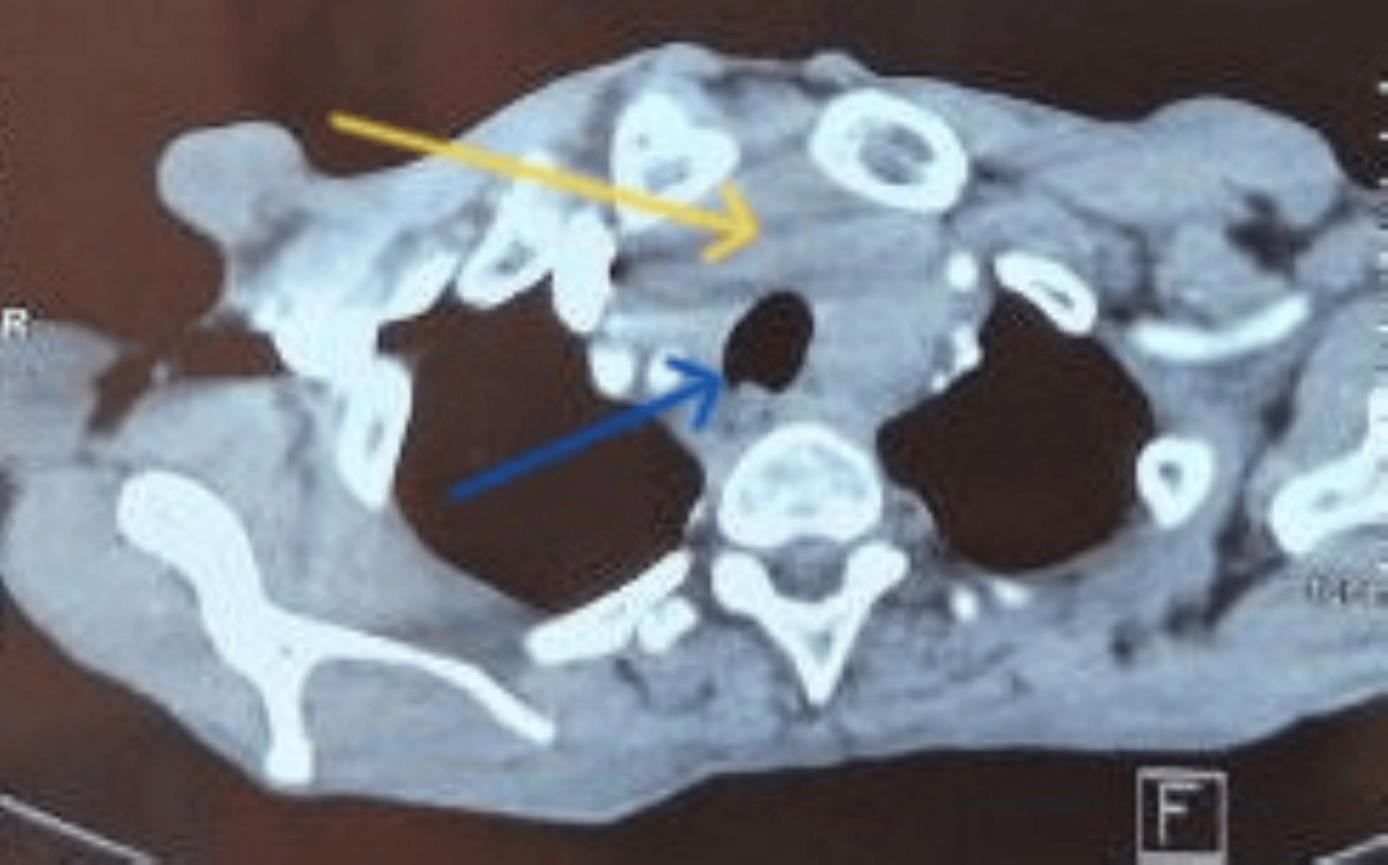
Cervicothoracic CT (axial view) showing a large substernal goiter (yellow arrow) causing a very mild deviation of the trachea (blue arrow)

This was further evaluated by cervicothoracic computed tomography (CT), which confirmed the presence of a plunging goiter without compression or invasion of the cervical vascular structures (Figure 2), although it induced a very slight deviation of the trachea (Figure 3). No clinically significant cervical lymphadenopathy was identified.

**Figure 4:**
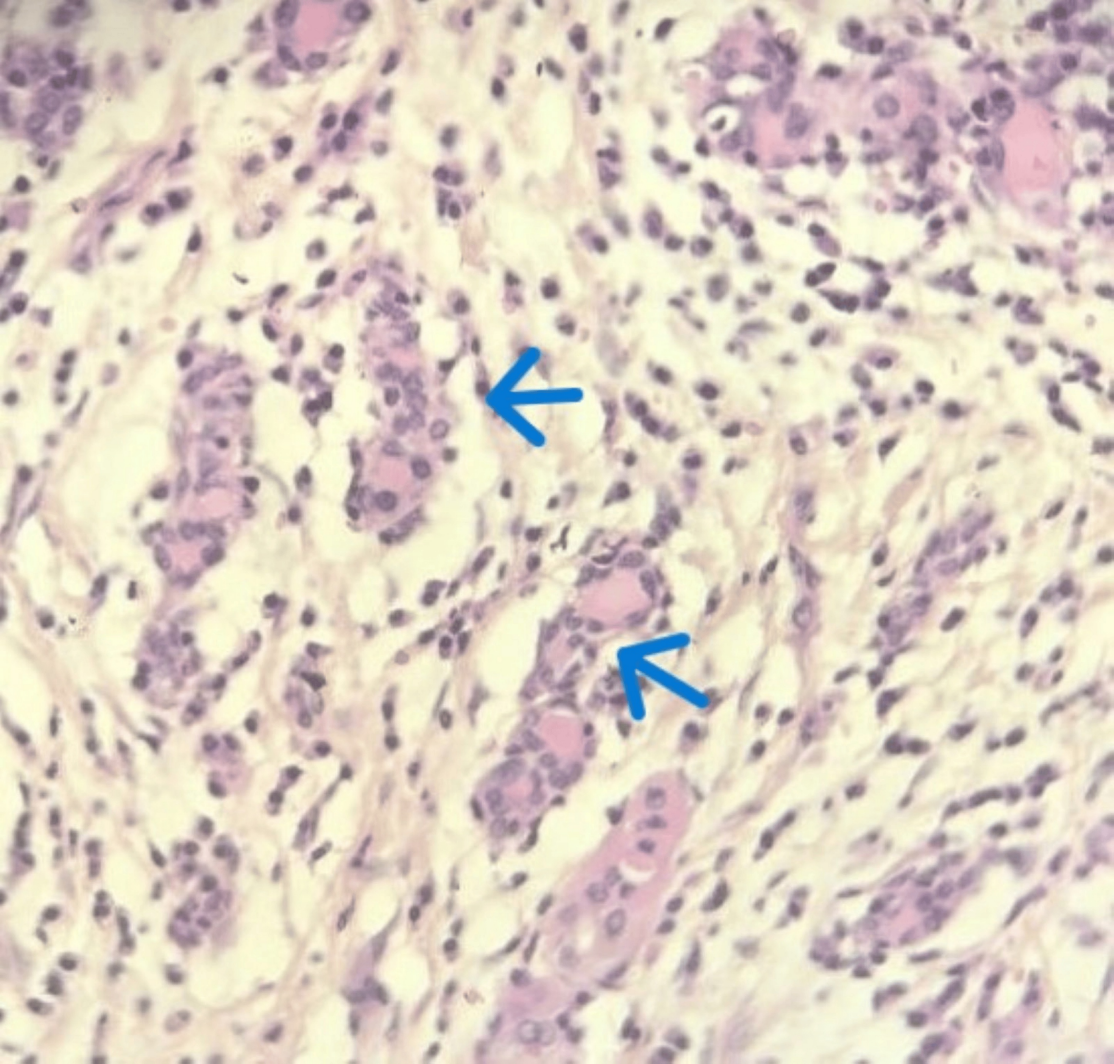
Microscopic image (40x) showing marked atrophy of the thyroid follicles (blue arrows).

**Figure 5:**
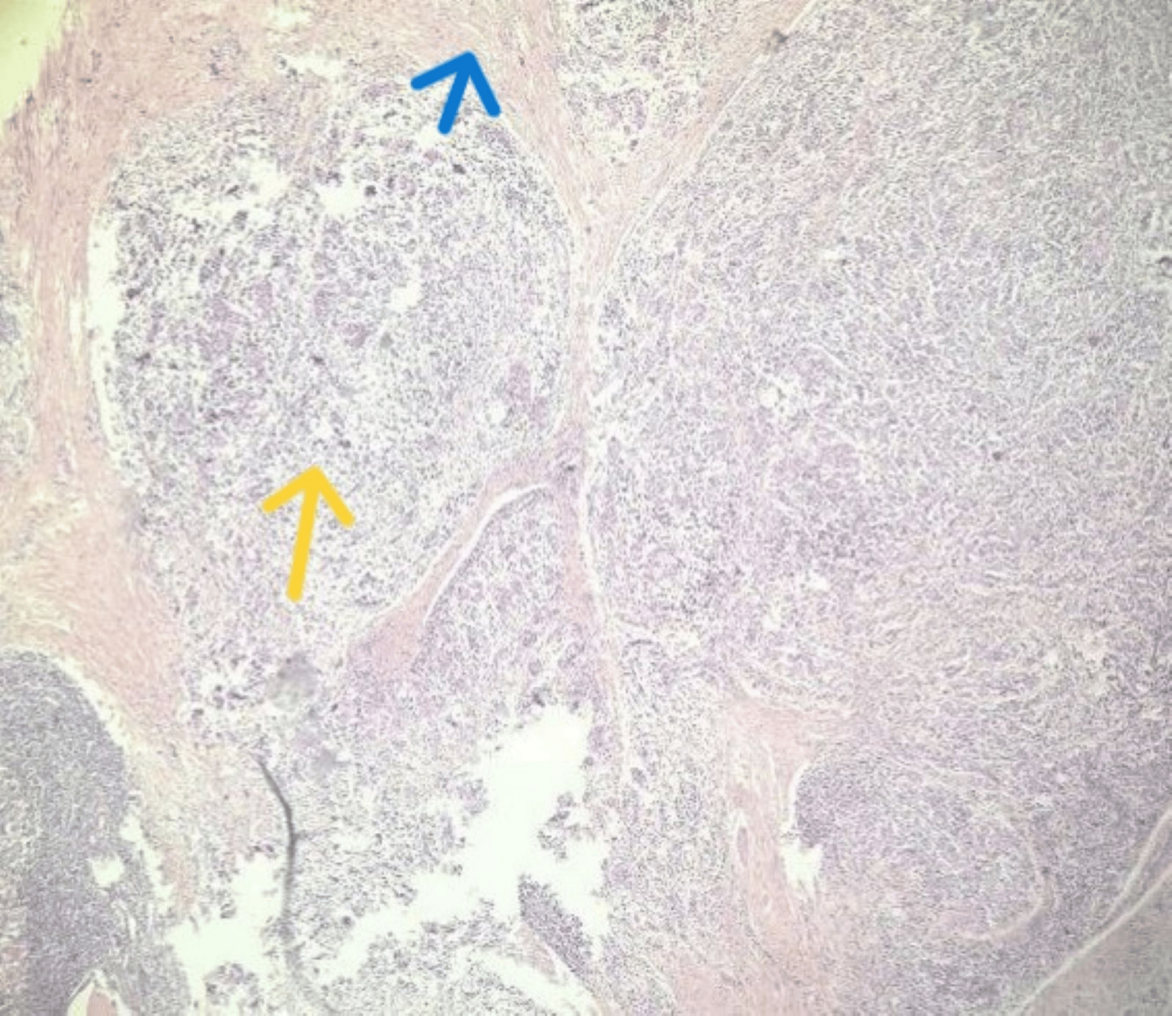
Microscopic image (H&E, 10x) reveals a diffuse destruction of the normal follicular architecture. The thyroid parenchyma is replaced by nodules of dense mononuclear inflammatory infiltrate forming secondary lymphoid follicles (yellow arrow), separated by fibrous septa (blue arrow).

**Figure 6:**
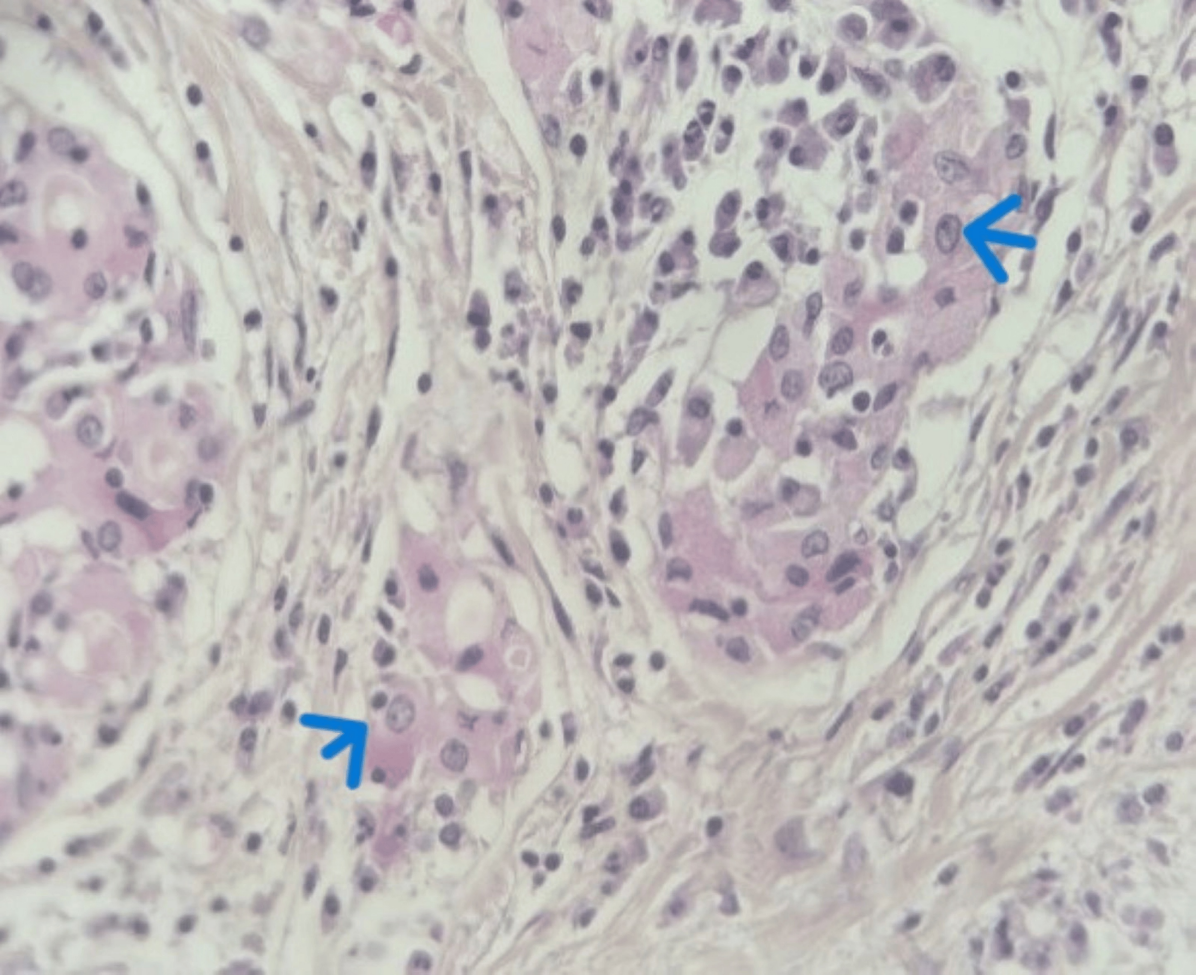
Microscopic image( 40x) showing follicular epithelial cells displaying oncocytic (Hürthle cell) change (blue arrow).

**Figure 7:**
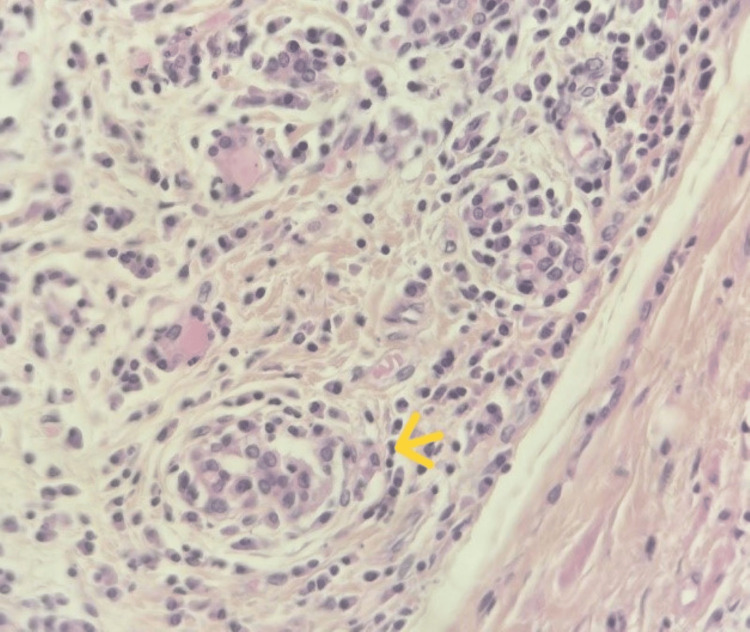
Microscopic image (40x) showing areas of squamous metaplasia (yellow arrow) within the thyroid tissue.

The patient subsequently underwent a total thyroidectomy. Macroscopically, the thyroid gland was enlarged, firm, and showed a whitish, homogeneous cut surface, conferring a pseudotumoral aspect. Histological examination revealed markedly atrophic thyroid tissue (Figure 4) with diffuse loss of the normal follicular architecture, largely replaced by extensive fibrous tissue. Within this fibrotic background, a dense and diffuse lymphoplasmacytic inflammatory infiltrate was observed, arranged in nodular aggregates separated by fibrous septa and forming secondary lymphoid follicles with well-developed germinal centers (Figure 5). Additionally, areas of oncocytic (Hürthle cell) metaplasia (Figure 6) and foci of squamous metaplasia (Figure 7) were identified. No cytological atypia or capsular or vascular invasion was noted. Taken together, these clinicopathological features are consistent with chronic autoimmune thyroiditis (Hashimoto’s thyroiditis), fibrosing variant.

**Figure 8:**
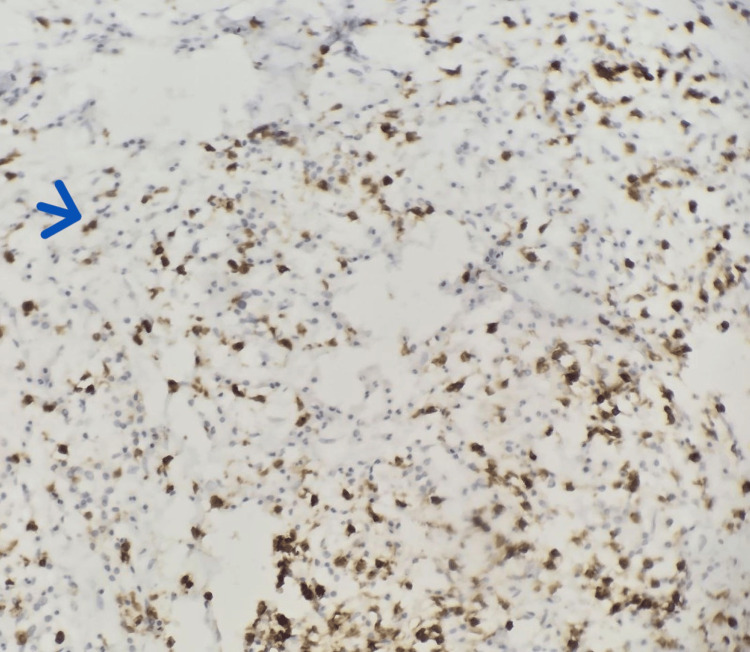
Immunohistochemical study performed using an anti-CD20 antibody. (10x) Staining highlights reactive lymphocytes (blue arrow).

**Figure 9:**
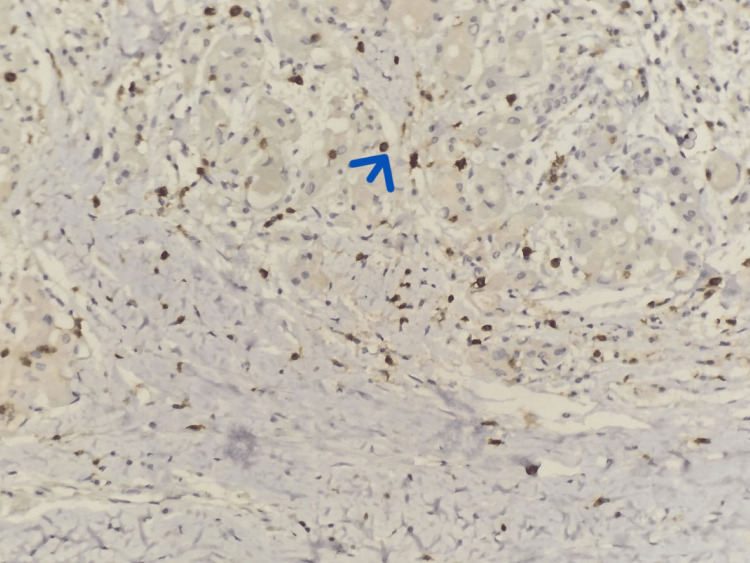
Immunohistochemical study performed using an anti-CD3 antibody.(10x) Staining highlights reactive lymphocytes (blue arrow).

An immunohistochemical analysis was performed using CD20 and CD3 antibodies (Figures 8, 9). The staining pattern was restricted to reactive lymphoid populations, without evidence of aberrant or diffuse monoclonal proliferation, thereby arguing against the diagnosis of lymphoma.

The immediate postoperative course was uneventful. The Redon drain was removed 24 hours after surgery, and postoperative serum calcium levels were within the normal range. The patient was discharged 48 hours following the procedure. Short- and mid-term follow-up showed a favorable outcome, with satisfactory wound healing and appropriate adjustment of thyroid hormone replacement therapy to levothyroxine 100 µg daily. Thyroid function tests performed three months after thyroidectomy were within normal limits.

## Discussion

Hashimoto’s thyroiditis is an autoimmune condition marked by gradual immune-mediated damage to thyroid follicles, arising from the interplay between genetic predisposition and environmental influences. It is the most common cause of hypothyroidism in populations exposed to excessive iodine intake [1]. An excessive iodine load can trigger or worsen hypothyroidism by causing a temporary suppression of iodine organification, a mechanism referred to as the Wolff-Chaikoff effect, from which an autoimmune thyroid gland is unable to adequately recover [2].

The fibrosing form represents a relatively rare subtype of Hashimoto’s thyroiditis, comprising roughly one-tenth of reported cases [3]. Patients typically present in mid-adulthood, with an average age of approximately 47 years, and the condition predominantly affects women, who account for nearly 94% of cases, corresponding to a female-to-male ratio of about 15:1 [4].

From a clinical standpoint, the fibrosing variant of Hashimoto’s thyroiditis follows a course that differs from the conventional form. Patients commonly present with a markedly hard goiter, frequently associated with a noticeable and relatively recent increase in gland size [5]. Manifestations may occur as the disease progresses. From a biochemical perspective, patients typically exhibit hypothyroidism accompanied by markedly increased levels of thyroid autoantibodies, notably anti-thyroglobulin and anti-thyroid peroxidase antibodies [6].

On macroscopic evaluation, the thyroid gland is often symmetrically enlarged during the initial stages, with a firm or nodular appearance and a volume reaching two to three times that of a normal gland. As the disease progresses, progressive atrophy may ensue, and in long-standing cases, only minimal or absent residual thyroid tissue and colloid can be observed [7].

At the microscopic level, the normal thyroid architecture is profoundly altered, with widespread fibrosis and significant follicular atrophy. A dense lymphoplasmacytic inflammatory infiltrate is present, frequently organized into germinal centers and intermingled with oncocytic cells. Areas of squamous metaplasia involving the follicular epithelium may also be identified. Notably, in this variant, the fibrotic process remains restricted to the thyroid gland [3]. This pattern differs from that observed in Riedel’s thyroiditis, where the fibrotic process is more aggressive, extending beyond the thyroid capsule and accompanied by a mixed inflammatory infiltrate composed of granulocytes, monocytes, and eosinophils, together with scattered plasma cells and CD8-positive T and B lymphocytes [8]. This lesion can be mistaken for a malignant neoplasm because metaplastic epithelial nests within the fibrotic stroma may closely resemble carcinomatous foci [5].

Certain thyroid malignancies, including papillary and anaplastic carcinomas, may exhibit marked fibrotic stroma, which can partially or completely mask the underlying neoplastic component as reported in the study by Baloch [9]. Furthermore, mucosa-associated lymphoid tissue (MALT) lymphoma exhibiting marked plasmacytic differentiation can closely mimic the fibrosing variant of Hashimoto’s thyroiditis [10].

## Conclusions

The fibrous variant of Hashimoto’s thyroiditis is a rare but clinically significant entity that can closely mimic malignant thyroid lesions both on examination and imaging. Its tumor-like presentation poses a diagnostic challenge, often raising concerns for cancer. Accurate diagnosis, therefore, hinges on meticulous histopathological evaluation, complemented when necessary by immunohistochemical studies. Heightened awareness of this variant is critical, as it allows clinicians and pathologists to avoid misdiagnosis and prevents patients from undergoing unnecessary aggressive treatments, ensuring care that is both precise and patient-centered.
